# Effort-Based Decision-Making and Gross Motor Performance: Are They Linked?

**DOI:** 10.3390/brainsci10060347

**Published:** 2020-06-04

**Authors:** Simone V. Gill, Samuel J. Abplanalp, Laura Keegan, Daniel Fulford

**Affiliations:** 1 Department of Occupational Therapy, Boston University, Boston, MA 02215, USA; samabp@bu.edu (S.J.A.); lakeegan@bu.edu (L.K.); dfulford@bu.edu (D.F.); 2 Department of Medicine, Boston University, Boston, MA 02215, USA; 3 Department of Psychology & Brain Sciences, Boston University, Boston, MA 02215, USA

**Keywords:** cognition, decision making, motor performance

## Abstract

The purpose of this study was to investigate the relationship between effort-based decision making and gross motor performance. Effort-based decision making was measured using a modified version of the Effort Expenditure for Rewards Task (EEfRT), in which participants pressed a button on a keyboard to fill a bar on a screen for monetary reward. Participants received monetary rewards that were commensurate with the level of effort that they were willing to expend. Gross motor performance was measured with a walking task, in which participants matched their steps to the beat of an audio metronome; they walked to metronome beats that were slower and also faster than their normal walking pace. We hypothesized that increased effort during the effort-based decision making task would be paired with an increase in steps taken per minute during the gross motor task. However, the results of this study indicated a lack of a statistically significant relationship between the effort-based decision making task and the gross motor task. Planning rather than decision-making may have been the cognitive construct that governed our gross motor task. These findings can be beneficial when thinking about potential interventions for populations who experience deficits in motor performance and cognition as well as for understanding the relationship between both cognitive and motor performance in healthy adults.

## 1. Introduction

Countless activities necessitate intact cognitive and motor processes [1,2,3]; for example, making it safely across a busy intersection requires deciding to cross during a gap in oncoming traffic and having the motor wherewithal to cross the street quickly. For impairments in cognition and motor performance, tasks requiring both processes pose a significant challenge to quality of life. 

On one hand, cognition and motor performance are linked due to shared neurobiological substrates [4,5,6,7]. For instance, the prefrontal cortex and the anterior cingulate gyrus have been implicated in decision-making and in monitoring actions [8]. Furthermore, the stages involved in decision-making are intertwined with motor performance (e.g., making a preference, executing an action, and experiencing an outcome) [8]. These steps suggest that cognitive (selection and planning) and motor (performing an action) processes [9] are inherent in decision-making, during which ongoing feedback guides action online [10,11,12].

On the other hand, the neurobiological substrates of decision-making and motor performance may be separate [13], as represented by traditional models and theories (i.e., dualism) [14,15,16,17]. The action execution step in decision-making may only refer to making a choice or a decision rather than executing a motor action. In such models, the neurological substrates implicated in decision-making may monitor taking action instead of gross motor execution [18,19].

Effort-based decision-making involves making a choice about whether a given outcome (typically a reward) is worth the mental or physical effort required to obtain it [20]. Previous studies have examined effort-based decision-making using the Effort Expenditure for Rewards Task (EEfRT) [21]. The EEfRT involves making a choice between either exerting physical effort for a monetary reward (i.e., money) via keyboard button pressing, or exerting no effort for less of a reward or no reward. The EEfRT has been used in studies of psychiatric populations, including people with schizophrenia and major depression, with results commonly showing that these groups demonstrate altered effort allocation by failing to make high-effort response choices that maximize reward. Prior research has hypothesized that deficits in effort-based decision-making may be due in part to motor impairments [22]; however, no study has examined this association using reliable, validated, and sensitive assessments of motor performance.

Motor performance is typically characterized by how well individuals perform tasks that have specified goals. For example, when asked to match their walking steps to the beat of an audio metronome, adults alter their steps based on the speed of the metronome; at paces 15% faster than their preferred walking pace, they increase the time at which their feet contact the ground [23,24,25]. Such modifications are indicative of intact motor performance because accuracy in matching the beat depends on walkers’ balance and coordination as they complete the task. Therefore, the metronome task is sensitive to changes in subtle motor functions. Measuring accuracy in timing walking steps is also sensitive to impairments in motor performance; older adults [26], adults with obesity [23], and adults with neurological impairments such as Parkinson’s Disease [27] demonstrate difficulty with timing their steps.

The purpose of this study was to examine the extent to which gross motor performance was related to effort-based decision-making. We tested effort-based decision-making using a modified version of the EEfRT, and motor performance with a task that involved matching walking steps to the sound of an audio metronome [24]. We hypothesized that gross motor performance (cadence) would be associated with a higher proportion of effortful trials chosen in the effort-based decision-making task. This hypothesis was based on recent studies supporting the possibility that such a link exists [28,29]. 

## 2. Materials and Methods

### 2.1. Ethics Statement

The study and consent procedures were approved by the Boston University Institutional Review Board and conformed to the Declaration of Helsinki. Informed written and verbal consent was obtained from all participants before testing began.

### 2.2. Participants

Fifty-nine participants responded to online or print flyers distributed in the Boston area or by word of mouth. Descriptive information about the participants can be found in Table 1A,B. As in our previous studies [24], potential participants were screened and excluded if they endorsed any of the following: below the age of 18 or above 65; had ever had a heart attack; ever been diagnosed with angina, asthma, cystic fibrosis, cardiovascular disease, bronchitis, obstructive lung disease, or a neurological or orthopedic condition; ever had a stroke; or if a physician had advised them against mild to moderate exercise. These exclusion criteria were in place because one of the primary tasks of the larger study included a vigorous exercise task [30].

### 2.3. Procedure

First, participants completed a metronome walking task (gross motor performance) followed by an effort-based decision-making task (cognitive). We assessed gross motor performance first to examine whether exerting physical effort would impact their responses during the cognitive task.

### 2.4. Metronome Walking Task

**Figure 1:**
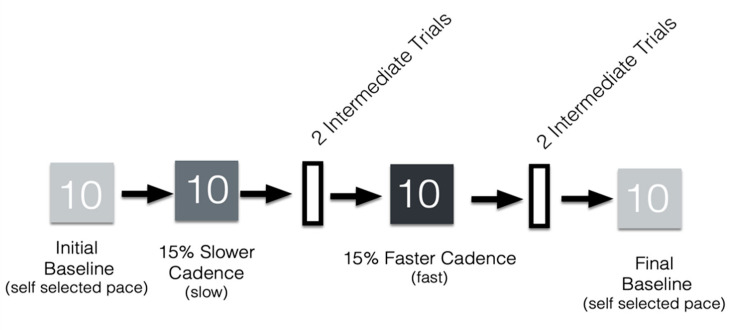
Metronome walking task procedure. The figure shows an example in which the slow metronome pace was played first. Light gray boxes represent initial and final baseline trials. Medium gray are slow metronome paces, and dark gray are fast metronome paces. After metronome trials at one pace were complete, participants walked for two intermediate trials at their own pace (white boxes).

Participants completed a gait task in which they were instructed to match their steps to an audio metronome beat as they walked along a 6.10 m × 0.89 m-long Protokinetics Gait Carpet (Protokinetics, LLC; Peekskill, NY, USA). They walked at their own pace and also to the beat of an audio metronome in 4 conditions: initial baseline, slow, fast, and final baseline (Figure 1). They first walked in an initial baseline condition that included 10 walking trials at their own pace (i.e., no metronome beat). For the slow and fast conditions, the average cadence (steps per minute) of each participant was calculated using measures from the initial baseline condition. Next, participants completed 10 trials in which the metronome beat was 15% slower than their baseline cadence, and 10 in which the beat was 15% faster. During the slow and fast metronome conditions, participants were asked to match their steps to the audio metronome by contacting the ground with their heels when the beat played. Participants then completed a final baseline condition for 10 trials at their own pace without the metronome beat. In between these trials, the participants completed 2 intermediately dispersed walking trials after each metronome condition at a self-selected pace (i.e., no metronome beat): Figure 1. Eight participants were excluded due to incomplete data from this task. 

### 2.5. Effort-Based Decision-Making Task

**Figure 2:**
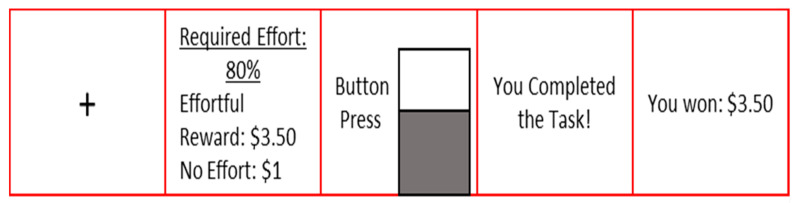
Effort-based decision-making task procedure. This is an example of an 80% effort trial with a reward of USD 3.50. Each trial began with the presentation of a fixation cross (Box 1). Participants then saw a Decision Prompt that prompted them to make a choice between the Effort Option presented (USD 3.50; Block 2) and the No Effort Option that always paid USD 1.00. They were required to make their selection within 3 seconds. They then used the keyboard to complete the task (Box 3). They were then told whether they completed the task (Box 4) and the reward amount won (Box 5).

We used a modified version of the EEfRT [21] as a measure of effort-based decision-making [31]; Figure 2. First, participants filled up a bar on a computer screen by tapping a computer key as quickly as possible using their non-dominant pinky finger. This procedure was completed three times to obtain a baseline measure of button-pressing speed. Varying amounts of monetary reward requiring different levels of baseline effort are displayed across a series of trials (Figure 2). The reward magnitude was shown as a dollar amount (range: USD 1–USD 5.73; based on four bins: USD 1.25–USD 2.39, USD 2.40–USD 3.49, USD 3.50–USD 4.60, and > USD 4.60), and the required effort level is shown by the height of a vertical bar (20%, 50%, 80%, or 100% of the participant’s maximum baseline button-pressing rate). Presented alongside each effortful option is the option of receiving USD 1 for no effort. There were 44 trials. An example trial may be a choice presented to either exert 80% of baseline effort expenditure for USD 2.45, or no effort for USD 1. These choices were hypothetical, given that participants were not required to exert effort in the moment. After making all of their choices, in another set of trials participants were then asked to either perform their selected choices or to reverse their initial decision and choose the non-effortful option for USD 1. One participant was excluded due to accepting every effortful choice. We chose to exclude this participant as the responses would provide no variability when conducting multilevel models.

### 2.6. Data Analysis

After excluding participants, we had a final sample size of (N = 50). The first level of analysis examined bivariate correlations between variables of interest: age, gender, condition (initial baseline, 15% slower, 15% faster), and proportion of effortful trials chosen from the effort-based decision-making task. Multilevel logistic models were used to examine associations between the outcome variable (i.e., proportion of effortful trial chosen) and within and between subject-level covariates using Hierarchical Linear Modeling [32] Version 7.03. We selected covariates for inclusion in the multilevel models based on our bivariate correlations and theoretical interest. The within-subject covariates were reward amount and effort level, stemming from the effort-based-decision-making task. The between-subject covariates were age, gender, initial baseline cadence, slow pace cadence (15% slower than the initial baseline cadence), fast pace cadence (15% faster than the initial baseline cadence). Parameters were estimated with full-information maximum likelihood and alpha levels were set at .05, as multilevel modeling uses partial pooling and provides more accurate estimates without the need to adjust for multiple comparisons [33]. 

## 3. Results

### 3.1. Bivariate Correlations

As shown in Table 2, the within-subject variable (i.e., proportion of effortful trials chosen) was not associated with any between-subject variables, including sex and age; however, there were trend-level associations between proportion of effortful trials chosen and baseline cadence (*r* = .27, *p* = .06), 15% slower cadence (*r* = .24, *p* = .093), and 15% faster cadence (*r* = .25, *p* = .08). Three within-subject variables were associated with one-another. Sex was negatively associated with all cadence variables, suggesting an association between identifying as female and a slower cadence in all walking conditions. 

### 3.2. Multilevel Models

In multilevel models, we included the binary outcome of effort or no effort selected across trials as the dependent variable. After running the baseline model, we then ran three models, each including reward amount and effort required as within-subject predictors (i.e., at Level 1), and age and sex as between-subject variables (i.e., at Level 2). Cadence levels (i.e., baseline, 15% slower pace, and 15% faster pace) served as additional between-subject variables in the three separate models. Each model also tested cross-level interactions, including the interaction between age and sex and effort level and reward amount. Cross-level interactions were also tested with all cadence variables, including the interactions between baseline, 15% slower pace, and 15% faster pace and effort level and reward amount. 

As shown in Table 3, reward amount and effort required were significant within-subject predictors of effortful trials chosen in all models. No between-subject variables, including all cadence variables, were significant predictors of effortful trials chosen. Furthermore, there were no significant cross-level interactions in predicting effortful trials chosen from the effort-based-decision-making task. 

## 4. Discussion

The purpose of this study was to investigate whether a relationship existed between effort-based decision-making and gross motor performance. Effort-based decision-making and gross motor performance were tested using an effort-based decision-making task and a walking task in which participants were asked to match their steps to an audio metronome. Inconsistent with our hypothesis, we identified no statistically significant relationship between effort-based decision-making and gross motor performance in any condition. Below, we propose three possibilities for our findings.

First, planning (conceiving of a strategy prior to carrying out an action) rather than decision-making may have been the cognitive construct that governed our gross motor task. Some researchers characterize planning as a part of decision-making in the context of gross motor performance [1,9,34,35,36]. However, for gross motor performance, decision-making may align with deciding whether or not to perform a task (i.e., “taking action”) or selecting from multiple ways to perform a task [9], rather than monitoring the performance of the task (i.e., matching steps to a metronome beat) [24]. Planning and decision-making may also have occurred serially rather than simultaneously [19,35,37] in our task: participants may have planned when to make initial heel contact prior to beginning the actual movement [14,15,16,17]. Thus, the neurological substrates involved in deciding on versus monitoring gross motor performance may be different [18,38,39,40]. Furthermore, our findings would suggest that the action execution referred to in the steps of decision-making is separable from executing gross motor action [11,41]. These findings also imply that among clinical populations, it may be more appropriate to assess planning and decision-making separately, prior to making assumptions about what contributes to impaired motor performance.

Second, while the EEfRT (and similar tasks) is considered to reflect cognitive decision-making, some researchers have conceptualized it as a test of physical effort, given the involvement of fine motor skills in rapid button pressing [42]. This task likely assesses the interaction of decision-making (as a cognitive process) and fine motor abilities. As such, the effort that a participant decides to exert may differ between fine and gross motor tasks [43]; using a hand versus a leg to complete a task may dictate the amount of effort exerted [44]. For example, adults report differences in their perception of distances [45], jumping height [46], and objects [18] based on the effector (e.g., leg or hand). Future research needs to be conducted to disentangle the relationships among decision-making in the context of exerting effort to perform fine versus gross motor tasks. 

Third, effort-based decision-making tasks offer rewards in relation to decisions that participants make, whereas our walking task provided no feedback about meeting the metronome pace. We intentionally omitted feedback to participants because we wanted to assess whether a relationship existed between effort-based decision-making and gross motor performance during a task that was purely focused on motor processes and because we thought that including the constraint to meet the metronome pace would transform walking into a complex, goal-oriented task [9,47] rather than the task of walking with no constraint [48,49,50]. It could be that effort-based decision-making and motor performance are more likely to intersect when an error in achieving a goal is perceived as in the introduction of a perturbation [9] or in relation to the cost of making an error [33,51]. Individuals’ motives may also interact with motor performance; specific types of incentives elicit motives and motives are related to tasks. For example, individuals may have implicit motives driven by achievement (satisfaction from mastering a task), affiliation (satisfaction in having positive relationships), or power (satisfaction from affecting others) [28]. It may be that each motive can only be elicited and lead to better motor performance if paired with the right incentive; those with a strong achievement motive may have performed better on the metronome task than those with either an affiliation or power motive. EEfRT offers explicit incentives (i.e., monetary reward) with outcomes that may or may not have matched participants’ motives during the metronome task. Future studies will examine possible links between decision-making and gross motor performance in the presence of a reward, motives, and with the option to decide whether or not to complete the walking trial based on metronome pace. Such an investigation may reveal the importance of applying a similar approach (i.e., testing the relationship between reward, motives, and motor performance) in clinical populations; for example, examining motives may allow clinicians to tailor cognitive and motor interventions to best meet patients’ needs. 

We acknowledge that our study has limitations. Although the same brain areas may be implicated in decision-making and motor performance, similarity in brain activation may not indicate similarity in mental states and functions. Furthermore, including a third task that tests both cognitive and motor performance may help to detect how each contributes to effort. Finally, we may have had restricted range in performance of our motor and decision-making tasks, given the relatively young, healthy sample. It is possible that relationships between gross motor performance and effort-based decision-making emerge in the context of impairment (e.g., limitations in motor performance may constrain decisions to exert effort).

Despite these limitations, our findings are relevant to an understanding of cognitive and motor processes in healthy adults. These results are also important for helping psychiatric populations who experience challenges in cognition and motor performance. Our study challenges the assumption that interventions targeting decision-making would also lead to improved gross motor performance and vice-versa. Thus, separate intervention approaches may be needed to improve decision-making and gross motor performance to enhance quality of life. However, this assumption should be taken with caution, because it is possible that this may not hold true in populations experiencing impairments in motor function, cognition, or both. This study helps to lay the foundation for investigations of motor and cognitive function. 

## Figures and Tables

**Table:** (**A**)

	Mean (%)
Age	23.74 (8.45; 18–65)
Gender	
Female	48.0
Male	52.0
Race	
Non-Hispanic Caucasian	62.0
Asian-American	30.0
African-American	4.0
Multiracial	2.0
Other not listed	2.0

**Table:** (**B**)

	Mean	Standard Deviation	Range
Cadence *			
Baseline	107.20	7.18	92–132
15% slower	91.12	6.43	76–112
15% faster	122.56	8.19	104–152
Effort based-decision making ^†^	0.71	0.12	0.43–0.93

* Steps per minute. ^†^ The proportion of effortful trials chosen across all trials.

**Table 2:** Bivariate correlations between demographic information, cadence, and effort-based decision-making (*N* = 50).

	1.	2.	3.	4.	5.
1. Age					
2. Gender	.24				
3. Baseline cadence	.15	−.33 *			
4. 15% slower cadence	.14	−.31 *	.97 **		
5. 15% faster cadence	.13	−.37 **	.98 **	.97 **	
6. Effort-based decision-making	.15	−.19	.27 ^†^	.25 ^†^	.24 ^†^

Notes: * *p* < .05; ** *p* < .01; ^†^ *p* ≤ .09.

**Table 3:** Within subject-level and between subject-level predictors of effortful choices (*N* = 50).

	Baseline Cadence Model	15% Slower Cadence Model	15% Faster Cadence Model
	Coefficient (SE) *p*	Coefficient (SE) *p*	Coefficient (SE) *p*
Within subject level			
Effort level	−6.49 (2.30) <.01	−6.69 (2.26) <.01	−6.66 (2.34) <.01
Reward amount	1.65 (0.42) <.01	1.65 (0.41) <.01	1.67 (0.43) <.01
Between subject level			
Age	0.03 (0.07) .64	0.04 (0.07) .60	0.03 (0.07) .63
Age X effort level	0.02 (0.01) .77	0.02 (0.08) .89	0.02 (0.08) .79
Age X reward amount	−0.01 (0.02) .88	−0.01 (0.01) .52	−0.01 (0.02) .54
Sex	0.40 (0.07) .74	0.29 (1.18) .81	0.35 (1.21) .77
Sex X effort level	−0.33 (1.46) .82	−0.20 (1.43) .89	−0.22 (1.48) .88
Sex X reward amount	−0.20 (0.26) .44	−0.21 (0.26) .43	−0.22 (0.27) .42
Baseline cadence	−0.03 (0.08) .68		
Baseline cadence X effort level	0.07 (0.10) .51		
Baseline cadence X reward amount	0.01 (0.02) .88		
15% slower cadence		−0.07 (0.10) .37	
15% slower cadence X effort level		0.12 (0.11) .31	
15% slower cadence X reward amount		0.01 (0.02) .92	
15% faster cadence			−0.03 (0.07) .64
15% faster cadence X effort level			0.07 (0.09) .43
15% faster cadence X reward amount			−0.01 (0.02) .98

## References

[1] Gill S.V., Hung Y.C. (2014). Effects of overweight and obese body mass on motor planning and motor skills during obstacle crossing in children. Res. Dev. Disabil..

[2] Gill S.V., Walsh M.K., Pratt J.A., Toosizadeh N., Najafi B., Travison T.G. (2016). Changes in spatio-temporal gait patterns during flat ground walking and obstacle crossing one year after bariatric surgery. Surg. Obes. Relat. Dis..

[3] Ogamba M.I., Loverro K.L., Laudicina N.M., Gill S.V., Lewis C.L. (2016). Changes in Gait with Anteriorly Added Mass: A Pregnancy Simulation Study. J. Appl. Biomech..

[4] Kayser A.S., Buchsbaum B.R., Erickson D.T., D’Esposito M. (2010). The functional anatomy of a perceptual decision in the human brain. J. Neurophysiol..

[5] Kayser A.S., Erickson D.T., Buchsbaum B.R., D’Esposito M. (2010). Neural representation of relevant and irrelevant features in perceptual decision making. J. Neurosci..

[6] Noppeney U., Ostwald D., Werner S. (2010). Perceptual decisions formed by accumulation of audiovisual evidence in prefrontal cortex. J. Neurosci..

[7] Philiastides M.G., Sajda P. (2007). EEG-informed fMRI reveals spatio-temporal characteristics of perceptual decision making. J. Neurosci..

[8] Ernst M., Paulus M.P. (2005). A selective review from a neurocognitive and clinical perspective. Biol. Psychiatry..

[9] Gallivan J.P., Chapman C.S., Wolpert D.M., Flanagan J.R. (2018). Decision-making in sensorimotor control. Nat. Rev. Neurosci..

[10] Scott S.H. (2004). Optimal feedback control and the neural basis of volitional motor control. Nat. Rev. Neurosci..

[11] Todorov E. (2004). Optimality principles in sensorimotor control. Nat. Neurosci..

[12] Todorov E., Jordan M.I. (2002). Optimal feedback control as a theory of motor coordination. Nat. Neurosci..

[13] Heekeren H.R., Marrett S., Ungerleider L.G. (2008). The neural systems that mediate human perceptual decision making. Nat. Rev. Neurosci..

[14] Glover S. (2004). Separate visual representation in the planning and control of action. Behav. Brain Res..

[15] Jeannerod M. (1990). The Neural and Behavioural Organization of Goal-Directed Movements.

[16] Rosenbaum D.A. (1991). Human Motor Control.

[17] Woodworth R.S. (1899). The accuracy of voluntary movement. Psychol. Rev..

[18] Filimon F., Philiastides M.G., Nelson J.D., Kloosterman N.A., Heekeren H.R. (2013). How embodied is perceptual decision making? Evidence for separate processing of perceptual and motor decisions. J. Neurosci..

[19] Kiani R., Shadlen M.N. (2009). Representation of confidence associated with a decision by neurons in the parietal cortex. Science..

[20] Treadway M.T., Zald D.H. (2011). Reconsidering anhedonia in depression: Lessons from translational neuroscience. Neurosci. Biobehav. Rev..

[21] Treadway M.T., Buckholtz J.W., Schwartzman A.N., Lambert W.E., Zald D.H. (2009). Worth the ‘EEfRT’? The effort expenditure for rewards task as an objective measure of motivation and anhedonia. PLoS ONE.

[22] Gold J.M., Waltz J.A., Frank M.J. (2015). Effort Cost Computation in Schizophrenia: A Commentary on the Recent Literature. Biol. Psychiatry.

[23] Gill S.V. (2015). Walking to the beat of their own drum: How children and adults meet task constraints. PLoS ONE.

[24] Gill S.V., Narain A. (2012). Quantifying the Effects of Body Mass Index on Safety: Reliability of a Video Coding Procedure and Utility of a Rhythmic Walking Task. Arch. Phys. Med. Rehabil..

[25] Gill S.V. (2015). The impact of weight classification on safety: Timing steps to adapt to external constraints. J. Musculoskelet Neuronal Interact..

[26] Mehmet H., Robinson S.R., Yang A.W.H. (2020). Assessment of Gait Speed in Older Adults. J. Geriatr. Phys. Ther. 2001..

[27] Corzani M., Ferrari A., Ginis P., Nieuwboer A., Chiari L. (2019). Motor Adaptation in Parkinson’s Disease During Prolonged Walking in Response to Corrective Acoustic Messages. Front. Aging. Neurosci..

[28] Muller F., Canal-Bruland R. (2020). Motivation in the wild: A critical review of the relationship between motives and motor performance. Motiv. Sci..

[29] Umesh A., Sanawu Kutten K., Hogan P.S., Ratnanather J.T., Chiib V.S. Motor cortical thickness is related to effort-based decision-making in humans. J. Neurophysiol..

[30] Abplanalp S.J., Fulford D. (2019). Physical effort exertion and pain: Links with trait-based risk for psychopathology. Psychiatry Res..

[31] Arulpragasam A.R., Cooper J.A., Nuutinen M.R., Treadway M.T. (2018). Corticoinsular circuits encode subjective value expectation and violation for effortful goal-directed behavior. Proc. Natl. Acad. Sci..

[32] Raudenbush S., Bryk A., Cheong Y., Congdon R. (2013). HLM for Windows.

[33] Gelman A., Hill J., Yajima M. (2012). Why We (Usually) Don’t Have to Worry About Multiple Comparisons. J. Res. Educ. Eff..

[34] Ben-Sasson A., Gill S.V. (2014). Motor and language abilities from early to late toddlerhood: Using formalized assessments to capture continuity and discontinuity in development. Res. Dev. Disabil..

[35] Dominguez-Zamora F.J., Marigold D.S. (2019). Motor cost affects the decision of when to shift gaze for guiding movement. J. Neurophysiol..

[36] Gill S.V. (2011). Optimising motor adaptation in childhood obesity. Aust. Occup. Ther. J..

[37] Johansson R.S., Flanagan J.R. (2009). Coding and use of tactile signals from the fingertips in object manipulation tasks. Nat. Neurosci..

[38] Andersen R.A., Buneo C.A. (2002). Intentional maps in posterior parietal cortex. Annu. Rev. Neurosci..

[39] Bennur S., Gold J.I. (2011). Distinct representation of a perceptual decision and the associated oculomotor plan in the monkey lateral intraparietal area. J. Neurosci..

[40] Gold J.I., Shadlen M.N. (2003). The influence of behavioral context on the representation of a perceptual decision in developing oculomotor commands. J. Neurosci..

[41] Faisal A.A., Selen L.P.J., Wolpert D.M. (2008). Noise in the nervous system. Nat. Rev. Neurosci. vol..

[42] Lopez-Gamundi P., Wardle M.C. (2018). The cognitive effort expenditure for rewards task (C-EEfRT): A novel measure of willingness to expend cognitive effort. Psychol. Assess..

[43] Gill S.V., Yang Z., Hung Y.C. (2017). Effects of singular and dual task constraints on motor skill variability in childhood. Gait Posture.

[44] Hagura N., Haggard P., Diedrichsen J. (2017). Perceptual decisions are biased by the cost to act. eLife.

[45] Proffitt D.R., Stefanucci J., Banton I., Epstein W. (2003). The role of effort in perceiving distance. Psychol Sci..

[46] Ramenzoni V.C., Riley M.A., Shockley K., Davis T. (2008). Carrying the height of the world on your ankles: Encumbering observers reduces estimates of how high an actor can jump. Q. J. Exp. Psychol..

[47] Nashed J.Y., Crevecoeur F., Scott S.H. (2012). Influence of the behavioural goal and environmental obstacles on rapid feedback responses. J. Neurophysiol..

[48] Gill S., Hicks G., Zhang Y., Niu J., Apovian C., White D.K. (2017). The association of waist circumference with community walking ability in knee osteoarthritis: The osteoarthritis initiative. Osteoarthr. Cartil..

[49] Lick D.J., Johnson K.L., Gill S.V. (2014). Why do they have to flaunt it? Perceptions of communicative intent predict antigay prejudice based upon brief exposure to nonverbal cues. Soc. Psychol. Personal. Sci..

[50] Lick D.J., Johnson K.L., Gill S.V. (2013). Deliberate Changes to Gendered Body Motion Influence Basic Social Perceptions. Soc. Cogn..

[51] Shadmehr R., Huang H.J., Ahmed A.A. (2016). A representation of effort in decision-making and motor control. Curr Biol..

